# Clinical Applications of Liquid Biopsy in Colorectal Cancer: A Focus on Registered Clinical Trials

**DOI:** 10.3390/genes17050500

**Published:** 2026-04-24

**Authors:** José Garcia-Pelaez, Yania Yáñez, Miguel Aupí, Marián Lázaro, Merche Molero, Miriam Oliver-Tos, Laura Rausell, Inés Calabria

**Affiliations:** Oncology Department, Health in Code Group, 46024 Valencia, Spain; yania.yanez@healthincode.com (Y.Y.); miguel.aupi@healthincode.com (M.A.); marian.lazaro@healthincode.com (M.L.); merche.molero@healthincode.com (M.M.); miriam.oliver@healthincode.com (M.O.-T.); laura.rausell@healthincode.com (L.R.)

**Keywords:** liquid biopsy, colorectal cancer, cell-free DNA (cfDNA), circulating tumor DNA (ctDNA), circulating tumor cells (CTCs)

## Abstract

**Background/Objectives**: Early detection through minimally invasive approaches is critical for timely patient stratification and optimal therapeutic decision-making in colorectal cancer (CRC). Liquid biopsy, based on the analysis of tumor-derived components in blood and other body fluids, has emerged as a promising strategy to overcome current limitations in CRC diagnosis and follow-up. This review evaluates the current landscape of liquid biopsy clinical trials in CRC, focusing on predictive biomarker detection, prognostic assessment, and disease monitoring. **Methods**: ClinicalTrials.gov was searched using the terms “colorectal cancer” and “liquid biopsy” yielding 153 registered trials. After manual screening, 44 trials were excluded for not using liquid biopsy for CRC management, leaving 109 trials for analysis. Of these, 25 were completed, and 13 had publicly available results related to liquid biopsy. **Results**: The included trials were conducted across 27 countries on four continents. Overall, 119 biomolecules assessments and 167 different endpoints were reported across 109 clinical trials. Because individual trials could evaluate multiple biomolecules and endpoints, counts exceed the total number of trials. Cell-free DNA (cfDNA) was evaluated in 92/109 trials (84%) and accounting for 77% of all biomolecule assessments. Circulatingtumor cells (CTCs) were analyzed in 9/109 trials (8%, representing 8% of all the biomolecules analyzed), and microRNAs (miRNAs) in 8/109 (7%, representing 7% of all the biomolecules analyzed). Treatment sensitivity was the most common endpoint (57/109, 52% of the clinical trials; representing 34% of all the 167 different endpoints analyzed), followed by disease progression (28/109, 26%; representing 17% of all the different endpoints analyzed) and diagnostic applications (21/109, 19%; representing 12% of all the different endpoints analyzed). Among the 25 completed studies, 10/25 (40%) were interventional and 15/25 (60%) observational, spanning 14 countries. The majority of completed trials (21/25, 84%) used cfDNA. Interventional studies were predominantly phase II (5/10), with fewer phase III trials (2/10), primarily evaluating treatment response, particularly in relation to EGFR inhibitors and RAS/BRAF mutation status. Four observational studies (4/15) investigated emerging biomarkers, including long noncoding RNAs and miRNAs. **Conclusions**: Current clinical trials highlight cfDNA as the dominant and most clinically advanced liquid biopsy biomarker in CRC, primarily used for treatment guidance and disease monitoring. In contrast, CTCs and RNA-based biomarkers remain underrepresented. The limited number of randomized late-phase trials, heterogeneity in study design, and technical challenges associated with emerging biomarkers underscore the need for standardized methodologies and robust validation before routine clinical implementation.

## 1. Introduction

### 1.1. CRC Incidence, Genetics and Pathogenesis

Colorectal cancer (CRC) is the fourth leading cause of cancer-related deaths worldwide, responsible for approximately 900,000 deaths annually [1], with incidence varying by region [2,3,4]. The prevalence of CRC is expected to rise, with an estimated 2.5 million new cases projected globally by 2035 [3,4]. Early-onset CRC is also increasing in an expanding number of countries [5]. Although genetic, lifestyle, and environmental factors are well-recognized contributors, the factors driving this rising incidence remain incompletely understood [6].

CRC can be classified into three categories based on molecular genetic similarities: chromosomal instability (CIN), microsatellite instability (MSI), and CpG island methylator phenotype (CIMP) [7,8].

CIN tumors, the largest subgroup, are characterized by aneuploidy, loss-of-heterozygosity (notably in *APC*, *DCC*/*MADH2*/*MADH4*, *TP53*), and mutations in *KRAS*, *BRAF*, and *SMAD4* [4,9].

MSI tumors, present in <10% of CRCs, result from defective DNA mismatch repair, and are often associated with Lynch syndrome [4,9].

CIMP tumors show promoter hypermethylation of multiple genes and may be microsatellite-stable or unstable, frequently harboring *RAS* or *BRAF* alterations that impact prognosis and therapy response [4,10,11].

Genome-wide studies have also found further markers in accordance with the mutation spectrum, such as the presence of *POLE*, *DCC*, *MYC* or *MCC* alterations [4,12,13,14].

Altogether, molecular characterization shows that CRC is a heterogeneous disorder. Although its genetics and key pathways are well studied, this knowledge has not yet led to effective methods for detection or non-invasive monitoring.

### 1.2. Management: Diagnosis and Treatment of CRC

CRC diagnosis relies on colonoscopy, imaging, and histopathological evaluation, including TNM staging and tumor subtyping [15]. In addition, tumor-based biomarkers are assessed, such as the *BRAF* V600E mutation, *RAS* mutations, HER2, *NTRK* gene fusions and the mismatch repair system (MMR) [16,17]. For instance, tumors with high microsatellite instability (MSI-H), deficient MMR (dMMR), or, in some cases, high tumor mutational burden (TMB) have important implications for clinical management, providing prognostic information and guiding treatment decisions, such as the use of immunotherapy in metastatic CRC, particularly in MSI-H/dMMR tumors [18,19,20,21,22].

Treatment options for colorectal cancer (CRC) typically include surgery, radiotherapy, chemotherapy, targeted therapies, and immunotherapy [23]. Management strategies differ depending on whether the tumor is resectable or metastatic, as surgical removal is not feasible in the majority of metastatic cases [17,18,24,25]. For resectable tumors, surgery may be combined with radiotherapy and/or chemotherapy in a (neo)adjuvant setting. In metastatic CRC, systemic treatments are preferred, including chemotherapy with agents such as fluoropyrimidines, oxaliplatin, and irinotecan, often combined with leucovorin. Targeted therapies are also part of the treatment for metastatic CRC. Bevacizumab, a humanized monoclonal antibody, inhibits angiogenesis by blocking vascular endothelial growth factor (VEGF) and is commonly combined with chemotherapy [17,21]. Cetuximab, an anti-EGFR monoclonal antibody, inhibits signaling pathways that regulate tumor-cell proliferation and survival. In *BRAF* V600E mutant CRC, combining cetuximab with a BRAF inhibitor such as encorafenib has shown significant clinical benefit [26]. Also, for those carrying the *KRAS* G12C variant, the combination of *KRAS* G12C inhibitors with EGFR monoclonal antibodies has shown to be useful [27]. For tumors with wild-type (wt) *KRAS*, panitumumab is preferred [28].

Additionally, approximately 5% of metastatic CRCs overexpress Human Epidermal Growth Factor Receptor 2 (HER2), and treatment with trastuzumab, combined with tucatinib, a selective HER2 inhibitor, has demonstrated encouraging efficacy [29].

Also, in <5% of metastatic CRCs, fusions in *NTRK* are found. However, testing is recommended when possible [17] to be eligible for treatment with TRK inhibitors (e.g., larotrectinib, entrectinib) [17,29].

As described previously, MSI-H/dMMR tumors, characterized by high mutational burden, are particularly responsive to immune checkpoint inhibitors (ICIs). Regarding this, examples of immunotherapies include pembrolizumab and nivolumab, which block PD-1, and ipilimumab, a CTLA-4 inhibitor [30].

Although these targeted therapies improved outcomes in specific molecular subgroups, treatment selection still largely depends on tissue sampling, which hampers dynamic evaluation of tumor evolution, such as, for instance, the emergence of resistance mutations (e.g., *KRAS* mutations acquired after an anti-EGFR therapy), since repeated biopsies are required to be properly identified [31].

Beyond these established systemic approaches, additional pharmacological strategies are being explored, including pharmacological agents targeting alternative tumor pathways or tumor microenvironment modulation, such as the use of sodium butyrate, which induces apoptosis in CRC cells [32] or nifedipine which showed effectiveness in inhibiting proliferation and metastasis in CRC by reactivating tumor immunity [33,34].

Taken together, this highlights the urgent clinical need for minimally invasive tools effective in evaluating tumor burden, predicting treatment response, and detecting recurrence, which could complement or eventually substitute conventional biopsy-based approaches.

### 1.3. Non-Invasive Detection of CRC

Early detection of CRC and its precursor lesions is essential for reducing disease-related mortality. Nevertheless, current screening strategies remain limited [35]. Stool-based tests such as fecal occult blood test (FOBT) and fecal immunochemical test (FIT) are used as first-line screening tools for CRC, with colonoscopy performed as follow-up after a positive result [36]. Serum tumor markers are not used for routine screening due to their very low sensitivity and specificity, particularly for early-stage or precancerous lesions, and are instead primarily used for monitoring treatment response, disease progression, and recurrence [25,35,36,37]. Liquid biopsy, detecting circulating tumor DNA, RNA, or other tumor-derived components in blood or other body fluids, emerges as a promising strategy to address these gaps [38,39,40]. Compared to tissue biopsy, liquid biopsy is faster, less invasive, and potentially allows for longitudinal monitoring of tumor dynamics and treatment response. However, standardization, clinical validation, and integration into existing workflows remain as the main challenges for its implementation [39].

The field of liquid biopsy is rapidly evolving as an efficient and non-invasive method for the detection, prognosis, treatment and recurrence of CRC, although still facing some challenges. Therefore, this review aims to analyze the current status as well as critically assess the landscape of liquid biopsy clinical trials in CRC, highlighting advances, limitations, and opportunities to implement this approach more effectively in clinical practice.

## 2. Current Status of Liquid Biopsy in CRC

### 2.1. Liquid Biopsy and Its Implications in Diagnosis and Treatment

Generally, non-invasive approaches such as imaging for breast cancer and screenings using stool or biofluids like blood and urine (e.g., in colorectal or prostate cancer) do not allow assessment of the genetic landscape of these malignancies. In this context, liquid biopsy has emerged as a minimally invasive strategy for molecular characterization, which can aid in accurate cancer diagnosis, patient stratification for targeted therapies, and monitoring of tumor evolution and treatment response over time. This approach provides a more comprehensive view of cancer heterogeneity and facilitates estimation of disease burden [38,41].

Over the past years, the field of liquid biopsy has evolved. Nowadays, it includes the detection of either circulating tumor cells (CTCs), circulating tumor DNA (ctDNA), extracellular vesicles (EVs), platelets modulated by cancer cells, proteins (such as carcinoembryonic antigen, CEA, in CRC), metabolites or cell-free RNAs, such as mRNA, long noncoding RNA (lnRNAs) and microRNA (miRNA), from different biofluids, with blood and stool being the most relevant in the context of CRC [38,42].

### 2.2. Circulating Tumor Cells (CTCs)

Recently, a large number of studies have concluded that the proportion of CTCs in the blood is associated with cancer development, especially during metastasis, confirming their implications as a relevant biomarker [43]. Thus, CTCs became an important tool for cancer diagnosis, especially shedding light on clinical decision-making [44,45,46]. Indeed, studies have demonstrated that higher levels of CTCs are linked with poor overall survival and progression-free survival [47,48]. However, due to their low concentration, sensitive tools are needed for their detection [49].

There are five different methods for CTCs characterization (reviewed in [43]), the main challenges to address are related with the sensitivity, specificity, time consumption and cost-effectivity [50,51,52,53]. The only method authorized by the FDA for the identification of the number of CTCs by the expression of specific markers in blood (by using antibodies combined with flow cytometry) is called CellSearch [15]. It is considered the gold-standard for CTC detection, and it is based on the epithelial cell adhesion molecule (EPCAM) expression [15,43].

Overall, for CRC, clinical data has already proved the prognostic involvement of CTCs at all stages of CRC [54], namely by using qPCR to detect mutated *KRAS*, *CK19*, *CK20* and *MUC2* [55]. On the other hand, the utility of CTCs in CRC screening or early detection is still limited [39].

### 2.3. Circulating Tumor DNA (ctDNA)

Blood samples may also contain cell-free DNA (cfDNA), fragments of DNA released into the bloodstream from normal and tumor cells. When the fraction of cfDNA derives specifically from tumor cells, it is known as circulating tumor DNA (ctDNA). This ctDNA provides a wider and comprehensive overview of tumor heterogeneity compared with a single biopsy of the tumor [56]. Currently, several methodological approaches are employed to identify actionable tumor biomarkers in plasma [57]. One strategy is a targeted approach that focuses on known genetic alterations in the primary tumor, typically within a limited panel of recurrent driver mutations with therapeutic relevance, such as *KRAS* or *EGFR* mutations [58]. The second strategy relies on an untargeted approach, conducted without prior knowledge of the specific genomic alterations present in the primary tumor. Genome-wide analysis of ctDNA enables the identification of tumor-specific alterations for disease monitoring, detection of resistance mechanisms, and the discovery of new therapeutic targets. A more cost-effective alternative to whole-genome sequencing is exome sequencing, which similarly does not require prior information about the tumor’s genetic landscape [56,57]. Technically, qPCR has been generally used to detect specific variants in ctDNA; however, due to the low concentration of it in early tumor stages, droplet digital PCR (ddPCR) has been preferably used to increase sensitivity and specificity [39]. Other approaches, including next-generation sequencing (NGS) and more sensitive PCR-based methods such as Beads-Emulsion-Amplification and Magnetics (BEAMing) or Amplification Refractory Mutation System (ARMS), are increasingly applied [59,60,61]. Supporting this, in a study in which a metastatic colorectal cancer cohort was used [62], cfDNA testing demonstrated distinct performance using different techniques: on the one hand, ddPCR achieved 47% sensitivity, 77% specificity, 70% positive predictive value (PPV), and 55% negative predictive value (NPV); on the other hand, BEAMing reached 93% sensitivity, 69% specificity, 78% PPV, and 90% NPV; while NGS showed 73% sensitivity, 77% specificity, 79% PPV, and 71% NPV, all relative to FFPE tumor tissue as reference. Regarding detection thresholds, ddPCR and NGS typically detect variants at 0.5–1% variant allele frequency (VAF) in cfDNA, while BEAMing can detect down to 0.03% VAF [62]. Overall, these results highlight the high sensitivity of BEAMing for low-frequency variants, whereas NGS provides a balanced performance across sensitivity and specificity relative to the other methods [62].

Also, epigenetic analysis is emerging as a novel approach to determine the methylation pattern in ctDNA due to its early detection capability and high specificity for different types of cancer [63].

The identification of genetic and epigenetic hallmarks in liquid biopsy has been applied to enable early detection of colorectal cancer (CRC), predict responses to specific therapies, and monitor disease recurrence in a minimally invasive manner (reviewed in [39]). *SEPT9* gene methylation has been reported as a non-invasive biomarker for early CRC diagnosis and disease surveillance, and is assessed through the FDA-approved Epi proColon test [64]. Additional biomarkers, such as *BCAT1* and *IKZF1* methylation—which are also useful for recurrence detection, as well as methylation of *APC*, *MGMT*, *RASSF2A*, and *WIF1* for early-stage CRC detection, have also been described [65,66]. Moreover, *NPY* methylation has been proposed as a potential biomarker for the early assessment of treatment response in metastatic CRC [67,68,69]. Additional blood-based methylation tests are already available to detect these signatures, namely the FDA-approved test called Shield, which attained an overall sensitivity over 80% for early detection of CRC [70]. Others, not yet approved by the FDA but still of relevance in the field of CRC, have been emerging such as COLVERA, which detects DNA methylation of *BCAT1* and *IKZF1* by real-time PCR, reaching an overall sensitivity of ≈60% for CRC recurrence [71], and the ColonAiQ assay, another multi-locus DNA-methylation assay for early detection of CRC at-risk individuals [72].

Prognostic biomarkers for early-stage colorectal cancer, including those associated with recurrence risk, include, for example, KRAS mutational status and *CDKN2A* hypermethylation [58]. Moreover, since the half-life of ctDNA is short (20–60 min), its levels decline rapidly following surgical removal of tumor tissue. Therefore, ctDNA can be used to monitor disease status, detect recurrence, and assess treatment response [39]. For instance, resistance to anti-EGFR or anti-HER2 therapies can be identified through the presence of *KRAS*, *BRAF*, and *NRAS* point mutations in ctDNA, as well as *ERBB2* (HER2 protein), *KRAS*, and *MET* amplifications, although the detection of such amplifications remains challenging [39,73]. Furthermore, ctDNA profiling has also been associated with other potentially relevant biomarkers, including *ROS1* fusions and alterations in *KRAS*, *NTRK1–3*, *RET*, *FGFR2–3*, and *ALK* [73].

### 2.4. Emerging Liquid Biopsy Analytes in Colorectal Cancer Management

In addition to circulating nucleic acids, other tumor-derived components obtained through liquid biopsy—such as extracellular vesicles (EVs)—have been evaluated as potential biomarkers. EVs released by tumor cells carry DNA, RNA (including microRNAs, miRNAs), and proteins [38]. Several EV-associated miRNAs have been reported to be enriched in the plasma of patients with colorectal cancer (CRC) compared with healthy controls, and their presence has been linked to early-stage disease, thereby enhancing the potential for early-stage CRC detection [74].

## 3. Registered Clinical Trials on Liquid Biopsy in Colorectal Cancer

### 3.1. Strategy to Search for Clinical Trials at ClinicalTrials.gov

**Figure 1:**
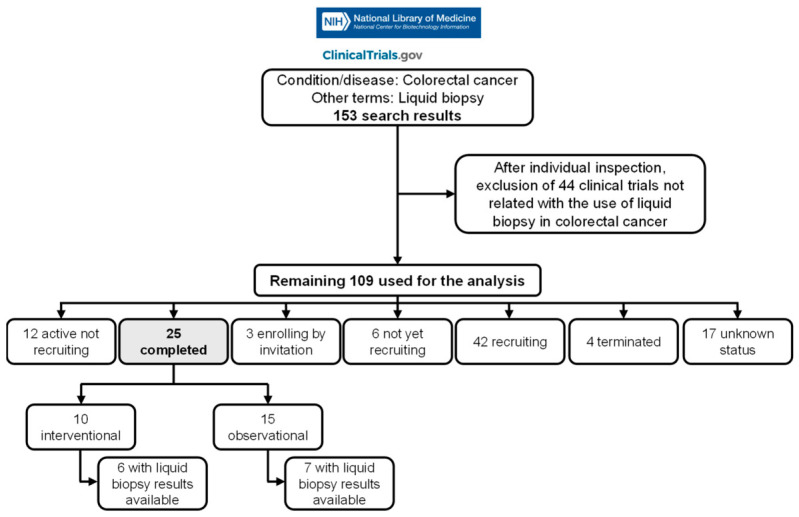
Flowchart describing the general strategy followed to search for clinical trials using liquid biopsy in CRC (source: https://clinicaltrials.gov/). In total, 109 clinical trials were used for analysis, of which 25 are already completed and 13 have available results related to liquid biopsy. Accessed on 29 September 2025.

The overall strategy followed is described in Figure 1. On ClinicalTrials.gov, a query for condition/disease “colorectal cancer” and other terms “liquid biopsy”, was performed (accessed on 29 September 2025). No filters were applied, and clinical trials were not restricted by recruitment status. Duplicates were checked based on NCT identifier and trial title; no duplicate entries were identified. This strategy retrieved 153 different registered clinical trials. Those using specific circulating biomarkers (e.g., cfDNA, ctDNA, CTCs, or miRNAs) but not explicitly registered as liquid biopsy trials, were not captured by this query and therefore may be underrepresented.

Out of the 153 clinical trials identified, 44 were excluded after manual verification because they did not explicitly report the use of liquid biopsy for CRC management. The remaining 109 were used for analysis and are described in detail in Supplementary Table S1. Of these 109 trials, 25 are already completed and 13 have results related to liquid biopsy available, either published in the literature or documented on ClinicalTrials.gov.

### 3.2. General Overview of Available Clinical Trials Using Liquid Biopsy for Colorectal Cancer

A total of 109 clinical trials were analyzed in detail, as shown in Table S1. They are being carried out in 27 countries from four continents (Supplementary Figure S1). The top three more represented countries are: the USA (where 20/109 [18%] clinical trials are being carried out: 4 interventional; 16 observational), Italy (with 17/109 [16%] clinical trials: 12 interventional; 5 observational) followed by China (with 10/109 [9%] clinical trials: 4 interventional; 6 observational). Among those completed (*n* = 25), 10 were interventional and 15 observational, distributed across 14 countries (Supplementary Figure S1). In total, 119 biomolecules evaluations and 167 different endpoints were reported across 109 clinical trials.

**Figure 2:**
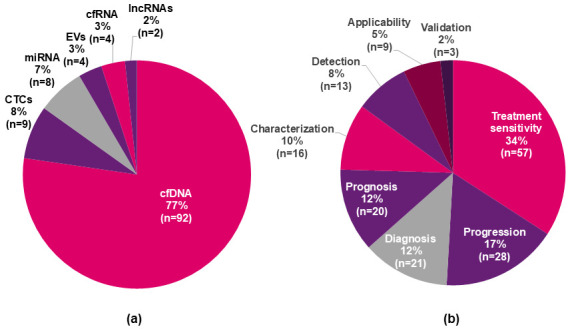
Landscape of available clinical trials using liquid biopsy for CRC. (**a**) Biomolecules assessed across 109 clinical trials. (**b**) Standardized endpoint categories of these clinical trials. Because some trials assess multiple biomolecules and endpoints, a single trial can be counted more than once across categories. CTCs, circulating tumor cells; lncRNAs, long non-coding RNA; cfDNA, cell-free DNA; cfRNA, cell-free RNA; EVs, extracellular vesicles; miRNA, microRNA.

92/119 (77%) of the biomolecules used to perform clinical trials in CRC patients were ctDNA/cfDNA (analyzed jointly in the same category); followed by CTCs, in 9/119 (8%); miRNA, in 8/119 (7%) and EVs, in 4/119 (3%), amongst other biomolecules that were represented in approximately 5% of them (Figure 2a). Regarding the endpoint overview of all the clinical trials, >50% of the outcomes were related to treatment sensitivity and disease progression (57/167 [34%] and 28/167 [17%], respectively); followed by diagnosis, in 21/167; (12%) and prognosis in 20/167 (12%) and characterization of tumor features in 16/167 (10%). A total of 9/109 of the clinical trials had the purpose of analyzing the applicability of liquid biopsy in the clinical context, including considerations regarding cost-effectiveness (Figure 2b).

### 3.3. Insights from Completed Clinical Trials

Twenty-five completed clinical trials were selected for further analysis (Table 1). As anticipated, the vast majority (21/25) of the clinical trials already completed used cfDNA. As previously referred, 10 were interventional and 15 observational. Among those classified as interventional, 5/10 are currently in phase II and 2/10 are already in phase III.

The primary objective of all five completed phase II studies was the evaluation of treatment sensitivity (NCT03227926; NCT03142516; NCT04425239; NCT04554836; NCT03829410), generally in the context of assessing the efficacy of EGFR inhibitors according to RAS/BRAF mutational status (Table 1 and Table S1). In contrast, the phase III studies were also focused on treatment sensitivity, but additionally addressed prognostic assessment in CRC patients and molecular profiling-based characterization (NCT02484833; NCT02934529).

Among the observational clinical trials, 2 of the 15 studies focused on CTCs to characterize the genomic landscape of metastatic CRC. Additionally, a research group in Egypt completed two observational trials investigating circulating miRNAs and lncRNAs (NCT03563651; NCT02809716), although results remain unavailable (Table 1). Together, these four studies underscore the continued exploration of emerging circulating biomolecule classes in CRC, including CTCs, miRNAs, and lncRNAs (Table 1 and Table S1).

### 3.4. Available Results on Completed Clinical Trials

Thirteen completed clinical trials have reported results, either in the literature or on ClinicalTrials.gov. Main characteristics, findings and data available from each completed trial are detailed below and in Supplementary Table S1.

**NCT03227926**

CHRONOS is an open-label, single-arm phase II clinical trial evaluating the feasibility of using liquid biopsy for detection of *RAS*, *BRAF*, and *EGFR* mutations in ctDNA to guide a chemotherapy-free rechallenge with panitumumab in patients with RAS wt metastatic CRC previously treated with anti-EGFR therapy. A total of 52 patients with tissue-confirmed RAS wt tumors were screened; 16 patients (31%) showed at least one resistance mutation in ctDNA, indicating ongoing molecular resistance to anti-EGFR therapy, and were therefore excluded. The remaining 36 patients were eligible, and 27 were enrolled and treated with panitumumab, achieving a 30% objective response rate (8/27 partial responses) and 63% disease control (17/27 including two unconfirmed responses). The study met its primary endpoint, demonstrating that ctDNA-guided selection can effectively and safely optimize anti-EGFR retreatment. However, limitations highlighted are the necessity to implement a larger panel of resistant variants in ctDNA to increase the effectiveness of anti-EGFR monoclonal antibodies, the risk of stochastic events that may affect the sensitivity and the fact that randomized trials are still missing (although already ongoing [75]). Moreover, they underscore the necessity to reduce operational and technical challenges [76].

**NCT06414304**

BLOOMSI is a prospective observational trial evaluating the impact of MSI/dMMR testing methods and baseline tumor heterogeneity on immunotherapy outcomes in 30 MSI/dMMR CRC patients. PCR/IHC testing and NGS of both formalin-fixed tissue (FFPE) and liquid biopsy were performed to assess concordance and mutation clonality. The objective response rate (ORR) in the intent-to-treat population was 50%, with concordance of 81% between local and central MSI/dMMR testing and around 68% among IHC, PCR, and NGS methods; however, patients with discordant results showed 0% ORR. Quantitative MSI analysis identified MSI clonality in FFPE and liquid biopsy as independent predictors of progression, suggesting that MSI heterogeneity may underlie resistance to immunotherapy; larger studies are needed to validate these findings. Although these data are preliminary, they indicate trends that will be further evaluated in the ongoing clinical trial [77].

**NCT04776837**

In total, 203 patients with metastatic gastrointestinal cancer (including CRC) were analyzed in this prospective study. Quality of life parameters were associated with treatment response and survival outcomes. A total of 160/203 completed the 1-month follow-up assessment. Functional Assessment of Cancer Therapy–General (FACT-G) scores over the first month of treatment were significantly associated with higher likelihood of clinical benefit, longer progression-free survival (PFS), and improved overall survival (OS), also analyzed by ctDNAs. Hence, although with limited results yet available, they conclude that quality of life parameters may serve as a useful biomarker for treatment response and survival in metastatic gastrointestinal cancers [78].

**NCT02792478**

PERSEIDA is a prospective, observational, multicenter study including 119 treatment-naive metastatic CRC patients to evaluate the concordance between tissue and liquid biopsies for *RAS* mutation detection. The study highlights the utility of BEAMing analysis, which identified additional *RAS* mutations in ctDNA, mostly at low mutant allele fractions (≥0.02%) [79]. Altogether, these findings underline the potential of liquid biopsy to guide treatment decisions and monitor therapeutic response over time. However, it is important to highlight that it was challenging to find statistically significant differences for some of the clinical outcomes (namely response rates or progression-free survival). Moreover, technically, for the second cohort they used, it shows the limitation of a less sensitive method [80]. Nevertheless, focusing on a homogeneous group of RAS wt patients enabled a more robust evaluation of liquid biopsy performance and mutation dynamics [81,82].

**NCT04425239**

This randomized study analyzed 137 patients with unresectable metastatic CRC (RAS/BRAF wt) divided into two groups: one receiving intermittent treatment and another one receiving continuous treatment based on conventional chemotherapy combined with an anti-EGFR therapy to assess overall response rates. Relevant clinical mutation in *KRAS*, *NRAS*, *BRAF*, *PI3K*, *EGFR*, *cKIT* and *PDGFR* genes to define potential biomarkers associated with disease activity and the efficacy or safety of treatment were analyzed. The main limitations of this study include: (i) the use of progression-free survival on treatment as the primary endpoint, which has not yet been clinically validated, and therefore it did not allow the comparison directly between the two arms, and (ii) the fact that not all randomized patients ultimately received the assigned post-induction strategy. Despite these limitations, this trial demonstrates that an intermittent FOLFIRI plus panitumumab strategy is feasible and achieves reduced toxicity while increasing time off treatment. Overall, the results are particularly encouraging for left-sided mCRC and may be strengthened further by ongoing translational studies aimed at improving patient selection [83].

**NCT05227261**

This prospective multicenter study evaluated the clinical utility of a multimodal, non-invasive, multi-cancer early detection (MCED) test based on methylation patterns and fragment size of ctDNA (SPOT-MAS). The study involved 9024 asymptomatic adult participants. Overall, the results revealed a positive predictive value of 39.5%, with a tissue of origin (TOO) accuracy of 52.94%, a negative predictive value of 99.9%, a sensitivity of 70.8% and a specificity of 99.7% across various cancer types. Out of 17 cases with true positive ctDNA signals, three cases were true positive for CRC (cases: K7706 [stage IVC CRC]; K4040 [stage IVC CRC]; K2409 [stage IIB CRC]); the three of them had biopsy positive results in the colon. However, three false negatives for CRC, and four for other cancers are also reported. False negatives are those diagnosed with cancer within 12 months of follow-up. Overall, the findings support the potential of ctDNA-based MCED testing to enhance early cancer detection and screening strategies. However, based on the observations for CRC, its detection appears lower than the overall pooled sensitivity, particularly for early-stage lesions, highlighting that early-stage CRC detection remains limited. The study also suggests that in general, a cost-effective multimodal liquid biopsy can support scalable early cancer detection and interception of precancerous disease in resource-limited settings, but its clinical impact remains constrained by limited case numbers, uncertain survival benefit, suboptimal performance in breast cancer, algorithmic and population-specific biases and the absence of direct comparison with established screening standards [84].

**NCT02484833**

The Phase III ERMES trial compared two maintenance strategies after first-line FOLFIRI + cetuximab in patients with RAS and BRAF wt metastatic CRC: continuing full FOLFIRI + cetuximab vs. switching to cetuximab monotherapy after induction. Cetuximab monotherapy reduces toxicity but fails to maintain equivalent disease control. Real-time ctDNA analysis enables early detection of clonal evolution under cetuximab maintenance, supporting decisions to discontinue anti-EGFR if resistant clones appear and consider EGFR rechallenge after mutation clearance. One limitation of the study was a high dropout rate due to the more fragile conditions of the patients included. Although noninferiority was not achieved with monotherapy, lower toxicity was demonstrated, so larger cohorts could help confirm a benefit in de-escalation [85].

**NCT02934529**

The FIRE-4 study randomly assigned patients with first-line RAS wt metastatic CRC to FOLFIRI plus cetuximab until progression or intolerable toxicity was observed, or to FOLFIRI plus cetuximab followed by a switch maintenance treatment using Fluorouracilplus bevacizumab. The study evaluates baseline liquid biopsy for detecting *RAS* and *BRAF* mutations in 540 metastatic CRC patients initially classified as RAS wt by tissue testing. *RAS* mutations were detected in 13% of cases and the V600E *BRAF* mutation in 7% of cases, showing worse survival outcomes and reflecting their true molecular status [86]. As an open-label clinical trial, a certain bias in outcome reporting is expected. However, the fact that this is a phase III randomized study and the fact that it had clinically meaningful survival rates, make these promising results [87].

**NCT04319354**

Although registered as interventional in ClinicalTrials.gov, the study design is prospective observational, since no experimental intervention was administered and patients received standard care. Thus, in this prospective observational study, cfDNA is investigated as a biomarker of response to neoadjuvant chemoradiotherapy (nCRT) in patients with locally advanced rectal cancer undergoing surgical excision. Serial liquid biopsies are collected at predefined time points (pre-nCRT, post-nCRT, and postoperative week 1) to assess cfDNA concentration, % of mutation frequency and mutational profile via NGS of tumor biopsies. Patients are stratified according to pathological response after surgery into complete, partial, and non-responders. Patients with higher baseline cfDNA whose levels decline progressively during and after nCRT may be more likely to achieve pathological complete response (PCR) and improved survival outcomes. This study demonstrates the feasibility of cfDNA-based monitoring as a non-invasive biomarker of treatment response in rectal cancer management. One of the limitations of the trial is the low number of patients recruited, so future trials involving a larger number of patients would be necessary [88].

**NCT04369053**

The PREEMPT CRC study is a prospective observational multicenter study evaluating a multiomics blood test for the early detection of CRC in participants aged 45 to 85 who are eligible for CRC screening and scheduled for a standard-of-care screening colonoscopy. For this purpose, they use *Freenome* which employs machine learning to identify patterns of cell-free biomarkers in blood for early cancer detection. Blood samples are collected prior to routine colonoscopy, and test performance is assessed against colonoscopy findings, with participants stratified by cancer, advanced adenoma, and non-advanced neoplasia. The study aims to evaluate the feasibility and clinical utility of multiomics blood testing as a non-invasive tool to support CRC screening, potentially enhancing adherence and facilitating earlier detection. However, it should be noted that the sensitivity of the test increases with the size of the lesion and the stage of the neoplasm [89].

**NCT03688906**

This study developed a blood-based, multiomic test using tumor- and immune-derived biomarkers to detect early-stage CRC in a cohort composed of 591 individuals. These findings show that combining multiomic signals can significantly enhance early CRC detection accuracy. This trial validated a multiomic liquid biopsy platform that integrates cfDNA whole-genome sequencing (WGS), bisulfite sequencing for epigenetic markers, and protein quantification to detect CRC. In a prospective cohort of 591 participants (including 548 colonoscopy-confirmed controls), the multiomic integration significantly outperformed individual assays, which achieved only 50–66% sensitivity. While the test demonstrated a high sensitivity of 92% for early-stage adenocarcinomas at 90% specificity, its performance was highly dependent on histological subtype. Notably, while it detected squamous cell carcinoma, it failed to identify neuroendocrine tumors, reducing overall sensitivity across all pathological subtypes to 80% in early stages. These findings suggest that while multiomic signals enhance detection for the majority of CRCs, pathological heterogeneity and the detection of rare subtypes remain significant challenges for blood-based screening tools [90].

**NCT04554836**

This study investigated dynamic changes in RAS mutational status among patients with initially *RAS*-mutated metastatic CRC during first-line therapy using BEAMing and ddPCR-based liquid biopsy assays. In 91% of patients exhibiting partial response or stable disease, *RAS* mutations in circulating tumor DNA converted to wt early during treatment, irrespective of chemotherapy regimen. These findings indicate that RAS conversion may identify a subset of patients who could benefit from anti-EGFR therapy despite initial RAS-mutant status. This study utilized high-sensitivity liquid biopsy assays (BEAMing and ddPCR) to perform an in-depth longitudinal monitoring of RAS mutational status in patients with initially RAS-mutant metastatic CRC (mCRC) during first-line therapy. The study revealed a high rate of molecular conversion, where 91% of patients with a positive therapeutic response (partial response or stable disease) saw their *RAS* mutations in ctDNA convert to wt (neoRAS-wt). This phenomenon occurred rapidly, with a median of 3.3–5.1 cycles, regardless of the chemotherapy regimen or anti-VEGF administration. To confirm that the disappearance of *RAS* mutations was due to clonal selection rather than low ctDNA shedding, the researchers used WIF1-promoter methylation as a secondary tumor marker, which remained detectable in 60% of cases after RAS conversion. These findings provide a biological rationale for the intermittent use of anti-EGFR therapy in initially RAS-mutant patients. However, the study is limited by its small longitudinal cohort (n = 20), which restricts the generalizability of these conversion rates. Furthermore, the clinical utility of this molecular window remains to be validated, as the study did not confirm whether rechallenging neoRAS-wt patients with anti-EGFR therapy translates into improved progression-free or OS [91].

**NCT03829410**

This is a phase II study involving 53 patients. They used a multicenter, open-label, single-arm study analyzing, for the first time, the safety and efficacy of a PLK1 inhibitor with a combination of conventional chemotherapies for patients with metastatic and unresectable CRC and a *KRAS* mutation in exons 2, 3, or 4. The detection of *KRAS*-mutant ctDNA was used for real-time assessment of tumor dynamics and treatment response. Thus, they found associations between *KRAS*-mutant ctDNA measures and ORR and PFS as well as confirming it as a sensitive and non-invasive pharmacodynamic biomarker [92]. As a previously raised concern, the fact that this is a single-arm study, with a limited number of cases highlights the necessity to develop larger and randomized controlled trials to further validate these results.

## 4. Additional Ongoing Clinical Trials on Colorectal Cancer Using Cell-Free DNA/Circulating Tumor DNA (cfDNA/ctDNA)

Since clinical trials using cfDNA have emerged as the most promising approach in the context of CRC, an additional, more comprehensive search of the literature was conducted to identify the most updated list of clinical trials not captured by the initial query on ClinicalTrials.gov.

Two multicenter, randomized clinical trials with results available have been registered in the Australian New Zealand Clinical Trials Registry (ACTRN12615000381583; ACTRN12617001566325). These studies, the latter designed as a phase II/III trial, evaluated stage II and stage III CRC cohorts, respectively, to assess recurrence risk and optimize adjuvant treatment strategies based on ctDNA analysis. In ACTRN12615000381583, a ctDNA-guided approach reduced the use of adjuvant chemotherapy without compromising recurrence-free survival (RFS). In contrast, ACTRN12617001566325 explored both treatment de-escalation and escalation strategies guided by ctDNA, demonstrating its utility as a strong prognostic biomarker; however, chemotherapy intensification in ctDNA-positive patients did not improve RFS, highlighting the need for alternative therapeutic strategies in this high-risk group [93,94].

Promising recent ongoing clinical trials for CRC management, not retrieved by the ClinicalTrials.gov query, are also exploring ctDNA-guided rechallenge strategies as study endpoints. This is the case for the randomized phase II CITRIC study, which evaluated rechallenge cetuximab and irinotecan by analyzing RAS/BRAF/EGFR-Extracellular domain mutations [95], supporting CHRONOS results. Other phase II trial representative examples are consistent with these results (namely, CRICKET [single arm, NCT02296203], VELO, [randomized, NCT05468892], or CAVE [single arm, NCT04561336]), reinforcing the role of ctDNA in guiding retreatment decisions. An in-depth description of ongoing clinical trials addressing this topic has been reviewed elsewhere [96].

## 5. Conclusions

In this study, we report a substantial number of registered clinical trials investigating the application of liquid biopsy for early detection, therapeutic monitoring, and molecular characterization of CRC. These initiatives are critical for the identification of robust biomarkers, the development of personalized treatment strategies, including immunotherapies, and ultimately for improving patient management and clinical outcomes.

Most of the currently ongoing clinical trials focus on cfDNA to detect CRC biomarkers in blood. However, other biomolecules seem to be emerging in the field of liquid biopsy for CRC diagnosis, treatment guidance and follow-up, namely CTCs, lncRNAs and miRNAs. However, no available results on these clinical trials are available yet and proven efficacy is needed in order to implement these approaches in the clinical setting. In addition, studying the expression by using lncRNAs and miRNAs, is still technically challenging [97].

One of the concerns extracted from this study and that may arise as a potential confounder in variants identification is the awareness of clonal hematopoiesis. Clinical trials like PERSEIDA (NCT02792478) report the identification of *RAS* mutations in ctDNA at a low VAF. Clonal hematopoiesis is relevant in this context since misattributing clonal hematopoiesis mutations as tumor-derived could misguide treatment decisions (i.e., RAS-targeted therapy eligibility). This underscores the need for cautious interpretation of ultra-sensitive liquid biopsy results.

Currently, only 4/25 completed clinical trials with results available in our ClinicalTrials.gov query have been randomized and had multiple arms, and therefore there is an enrichment in single-arm and non-randomized clinical trials. Therefore, this limits the solid applicability of liquid biopsy for CRC management into the clinics. Overall, our study underscores the need to refine clinical trial design, including the incorporation of larger cohorts, to generate more robust and consistent results.

The majority of registered clinical trials in ClinicalTrials.gov included in this study, as well as those most recently completed with available results, underscore the utility of liquid biopsy in assessing treatment response, typically in the context of evaluating EGFR inhibitors according to *RAS*/BRAF mutation status and in guiding therapy rechallenge strategies in patients previously treated with anti-EGFR agents (namely, CHRONOS (NCT03227926), FIRE-4 (NCT02934529), CAPRI 2 GOIM (NCT05312398), CAVE 2 GOIM (NCT05291156), found in Table S1). These findings further support its promising application in the clinical setting. Indeed, current ESMO clinical guidelines for metastatic CRC recommend that cfDNA analysis from plasma be considered to determine RAS mutation status when adequate tissue is unavailable, thereby facilitating optimal treatment selection [17,98,99,100].

Cancer progression is the second most common endpoint among all the clinical trials analyzed on ClinicalTrials.gov. Regarding this, both the ESMO clinical guidelines for localized colon cancer [25] and the NCCN guidelines for colon cancer (version 5.2025) [101], highlight the utility of liquid biopsy to determine the risk of recurrence. Nonetheless, for both therapeutic purposes and to clarify relapse status, the ESMO guidelines underline that it must be awaited before these can be accepted in routine practice [19,25,102]. In fact, after briefly analyzing the main endpoints retrieved by a secondary query on ClinicalTrials.gov based solely on cfDNA in the context of CRC, we observed that the retrieved trials were predominantly focused on recurrence. This highlights a limitation of our primary search strategy on ClinicalTrials.gov: because terminology in trial registries is not standardized, some studies analyzing specific circulating biomarkers, such as cfDNA, ctDNA, CTCs and miRNAs, may not have been captured if the term “liquid biopsy” was not explicitly included in the trial registration. Nevertheless, restricting our search to trials that explicitly use the term “liquid biopsy” allowed us to evaluate how this concept is currently framed and implemented in clinical trial design and reporting. However, to overcome this limitation, a more comprehensive review of cfDNA clinical trials in the context of CRC is presented in Section 4. Nonetheless, future studies should complement our approach by systematically exploring biomarker-specific terminology to better characterize the clinical utility and evolution of individual biomolecules used in liquid biopsy.

To the best of our knowledge, only 5/109 clinical trials in our ClinicalTrials.gov query (NCT06989814; NCT06163365; NCT06708429; NCT06726642; NCT04261972) are devoted to the application of liquid biopsy in Lynch syndrome patients, which are at high risk of CRC development at an early age of onset [103,104]. Only one more (early-stage) clinical trial (NCT06218433) appears when we perform a query on ClinicalTrials.gov about this subject (condition/disease “Lynch Syndrome” and other terms “liquid biopsy”). Multicenter initiatives such as the European project “predi-Lynch” may shed light on this gap, since its aim is to develop and validate non-invasive, accurate, and cost-effective liquid biopsy tests to detect cancer at its earliest stages in those carrying germline variants in Lynch syndrome-associated genes [105]. This would be crucial to develop strategies to prevent CRC in young patients with other rare tumor-risk syndromes, such as the PTEN Hamartoma Tumor Syndrome (PHTS) [106,107], and improve their pathway of care, with economic and social impacts, as claimed, for instance, by the European project PREVENTABLE [108].

Altogether, this analysis allowed us to analyze how the trajectory of liquid biopsy development in CRC differs from that observed in other solid tumors, such as lung cancer, where early adoption was facilitated by highly actionable genomic alterations and rapid therapeutic targets [109,110]. Indeed, for other cancer types, such as breast, lung or gastrointestinal cancers, previous recent large cohorts studies have demonstrated a high specificity and positive predictive values when assessing ctDNA liquid biopsy in comparison with tissue-based PCR/NGS testing as well as a high accuracy for single nucleotide variants (SNVs), conferring reliable evidence with strong potential to be implemented in the clinical setting [109,110,111,112,113,114]. For instance, in breast cancer, it has been demonstrated that using liquid biopsy for a tumor’s genotyping provided more accuracy than standard serum markers [61]. In contrast, for CRC, the limited spectrum of immediately actionable mutations and the biological complexity of tumor heterogeneity may partly explain the slower transition toward routine clinical use.

One potential future direction in ctDNA use for CRC lies in the neoadjuvant setting: for example, immunotherapy trials such as NCT04165772 have shown promising results in dMMR CRC, highlighting the potential for integrating molecular biomarkers, including ctDNA, for treatment stratification [115]. Additionally, ongoing clinical trials (Table S1) such as SAGITARIUS (NCT06490536) and PEGASUS (NCT04259944) remark the use of ctDNA to guide adjuvant therapy strategies [116,117].

In conclusion, our work provides an overview of the current landscape of clinical trials in the field, summarizing their scope and relevance as well as unveiling potential gaps to be addressed in future studies. Altogether, the observations reported herein will help clarify the context for the effective clinical application of liquid biopsy in CRC.

## Figures and Tables

**Table 1:** List of 25 completed clinical trials with a brief description and availability of results.

NCT Number	Sample Source	Endpoint Category	Liquid Biopsy Type	Results in ClinicalTrials.gov?	Publication	Study Type	Country
NCT03563651	Blood/urine/stool	Detection/ Characterization	CTCs	No results	-	Observational	USA
NCT06432413	Blood	Prognosis	lncRNAs/miRNAs	No results	-	Observational	Egypt
NCT03227926	Blood	Treatment sensitivity	cfDNA	No results	PMID: 35915157	Interventional (Phase II)	Italy
NCT04566614	Blood	Treatment sensitivity/Prognosis	cfDNA	No results	-	Observational	UK
NCT06414304	Blood	Treatment sensitivity	cfDNA	No results	PMID: 40244273	Observational	Russia
NCT05875584	Blood	Diagnosis	cfDNA	No results	-	Observational	China
NCT04104633	Blood	Characterization/Applicability	cfDNA	No results	-	Interventional	France
NCT04776837	Blood	Progression	cfDNA	No results	DOI: 10.1200/JCO.2022.40.16_suppl.6570	Observational	USA
NCT02792478	Blood	Detection/Treatment sensitivity	cfDNA	No results	PMID: 36551560; 38642257	Observational	Spain
NCT03142516	Blood	Treatment sensitivity/ Progression	cfDNA	No results	-	Interventional (Phase II)	Spain
NCT06427278	Blood	Progression/Prognosis	lncRNAs/miRNAs	No results	-	Observational	Egypt
NCT04425239	Blood	Applicability/ Treatment sensitivity	cfDNA	No results	PMID: 39576946	Interventional (Phase II)	Italy
NCT06531902	Blood	Diagnosis/Prognosis	cfDNA	No results	-	Observational	Egypt
NCT05227261	Blood	Diagnosis	cfDNA	No results	PMID: 39948555	Observational	Vietnam
NCT06738511	Blood/stool	Applicability/Characterization	cfDNA	No results	-	Interventional	Germany/Poland
NCT02595645	Blood	Detection	cfDNA	No results	PMID: 29511559	Interventional	Germany/Austria
NCT02809716	Blood	Applicability/Characterization	CTCs	No results	-	Observational	USA
NCT02484833	Blood	Treatment sensitivity/Characterization	cfDNA	No results	PMID: 38181312	Interventional (Phase III)	Italy
NCT02934529	Blood	Treatment sensitivity/Prognosis	cfDNA	No results	PMID: 39903881	Interventional (Phase III)	Germany
NCT04319354	NS	Treatment sensitivity	cfDNA	No results	PMID: 36986526	Observational	Portugal
NCT04369053	Blood	Diagnosis	cfDNA	No results	PMID: 40207404	Observational	USA
NCT03688906	Blood	Diagnosis	cfDNA	No results	DOI: 10.1200/JCO.2020.38.4_suppl.66	Observational	USA/ Canada
NCT04554836	Blood	Treatment sensitivity	cfDNA	Results available	-	Interventional (Phase II)	Germany
NCT05697198	Blood	Characterization/Treatment sensitivity	cfDNA	No results	-	Observational	USA
NCT03829410	Blood	Treatment sensitivity	cfDNA	Results available	PMID: 39475591	Interventional (Phase I/II)	USA

NS, not specified; CTCs, circulating tumor cells; lncRNAs, long non-coding RNA; cfDNA, cell-free DNA; miRNA, microRNA; UK, United Kingdom; USA, United States of America.

## Data Availability

All data used to generate this manuscript are publicly available at ClinicalTrials.gov.

## Supplementary Materials

The following supporting information can be downloaded at: https://www.mdpi.com/article/10.3390/genes17050500/s1, Table S1: Detailed description of clinical trials using liquid biopsy for colorectal cancer; Supplementary Figure S1: Global distribution of 109 clinical trials applying liquid biopsy in colorectal cancer (CRC).

## Abbreviations

The following abbreviations are used in this manuscript:

ARMS	Amplification Refractory Mutations System
BEAMing	Beads-Emulsion-Amplification and Magnetics
CEA	Carcinoembryonic antigen
cfDNA	Cell-free DNA
CIMP	CpG island methylator phenotype
CIN	Chromosomal instability
CRC	Colorectal cancer
CT	Computed tomography
CTCs	Circulating tumor cells
ctDNA	Circulating tumor DNA
ddPCR	Droplet digital PCR
dMMR	Deficient mismatch repair
EGFR	Epidermal growth factor receptor
EVs	Extracellular vesicles
FACT-G	Functional assessment of cancer therapy-general
FFPE	Formalin-fixed paraffin-embedded
GFP	Green fluorescent protein
HER2	Human epidermal growth factor receptor 2
ICIs	Immune checkpoint inhibitors
IHC	Immunohistochemistry
lnRNA	Long noncoding RNA
MCED	Multi-cancer early detection
miRNA	microRNA
MMR	Mismatch repair
MRI	Magnetic resonance imaging
MSI/MSI-H	Microsatellite instability
NGS	Next generation sequencing
nCRT	Neoadjuvant chemoradiotherapy
NPV	Negative predictive value
ORR	Objective response rate
OS	Overall survival
PCR	Polymerase chain reaction
pCR	Pathological complete response
PFS	Progression-free survival
PHTS	PTEN Hamartoma Tumor Syndrome
PPV	Positive predictive value
RFS	Recurrence-free survival
SNVs	Single nucleotide variants
PD-1	Programmed death-1
TMB	Tumor mutation burden
TOO	Tissue of origin
VAF	Variant allele frequency
VEGF	Endothelial growth factor
WT	Wild type
