# Supplementary material for: Clinical Applications of Liquid Biopsy in Colorectal Cancer: A Focus on Registered Clinical Trials
Source: Genes (Basel). 2026 Apr 24;17(5):500. doi: 10.3390/genes17050500 (PMC13205975; doi:10.3390/genes17050500)
Supplement: Supplementary file 1 [file genes-17-00500-s001.zip › Supplementary figure S1.pdf]

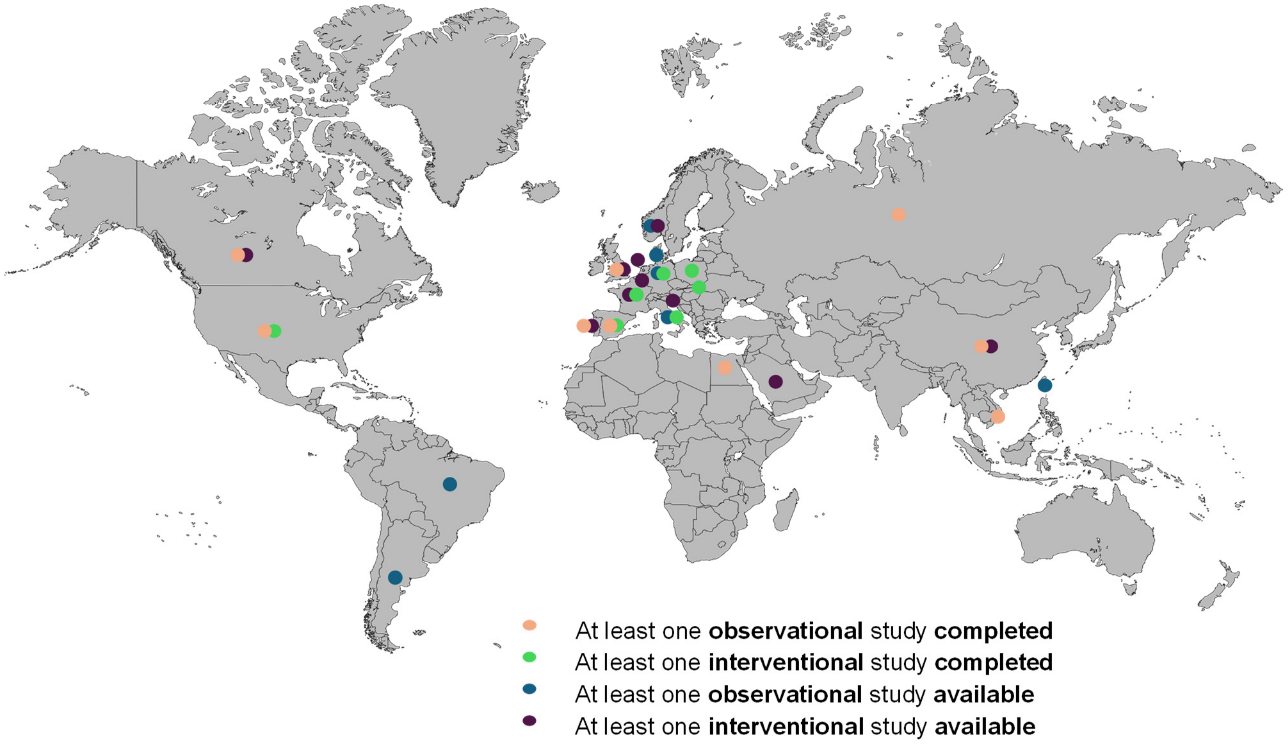

Supplementary Figure S1. Global distribution of 109 clinical trials applying liquid biopsy in colorectal cancer (CRC). Purple and blue dots indicate countries conducting at least one interventional or observational study, respectively. Countries with at least one interventional or observational study completed were marked in green and orange respectively (instead of pink and blue). Map template retrieved from BioRender.com.
